# Impact of Doping on Cross-Sectional Stress Assessment of 3C-SiC/Si Heteroepitaxy

**DOI:** 10.3390/ma16103824

**Published:** 2023-05-18

**Authors:** Viviana Scuderi, Marcin Zielinski, Francesco La Via

**Affiliations:** 1 Consiglio Nazionale delle Ricerche, Istituto per la Microelettronica e Microsistemi (CNR-IMM), Ottava Strada n.5, 95121 Catania, Italy; francesco.lavia@imm.cnr.it; 2 NOVASiC, Savoie Technolac, 73375 Le Bourget du Lac, France; mzielinski@novasic.com

**Keywords:** 3C-SiC, doping, Raman analysis

## Abstract

In this paper, we used micro-Raman spectroscopy in cross-section to investigate the effect of different doping on the distribution of stress in the silicon substrate and the grown 3C-SiC film. The 3C-SiC films with a thickness up to 10 μm were grown on Si (100) substrates in a horizontal hot-wall chemical vapor deposition (CVD) reactor. To quantify the influence of doping on the stress distribution, samples were non-intentionally doped (NID, dopant incorporation below 10^16^ cm^−3^), strongly n-type doped ([N] > 10^19^ cm^−3^), or strongly p-type doped ([Al] > 10^19^ cm^−3^). Sample NID was also grown on Si (111). In silicon (100), we observed that the stress at the interface is always compressive. In 3C-SiC, instead, we observed that the stress at the interface is always tensile and remains so in the first 4 µm. In the remaining 6 µm, the type of stress varies according to the doping. In particular, for 10 μm thick samples, the presence of an n-doped layer at the interface maximizes the stress in the silicon (~700 MPa) and in the 3C-SiC film (~250 MPa). In the presence of films grown on Si(111), 3C-SiC shows a compressive stress at the interface and then immediately becomes tensile following an oscillating trend with an average value of 412 MPa.

## 1. Introduction

Its good mechanical and electrical properties make silicon carbide (SiC) a material of interest both in electronic devices (sustainable energy, hybrid vehicles, low-power loss inverters) [1,2] and in sensor applications (micro electromechanical systems (MEMS)) [3].

The reason for the development of a technology based on 3C-SiC heteroepitaxy mainly lies in the possibility of reducing the costs of production. However, there are some open issues for a complete development of high-performance devices based on heteroepitaxial 3C-SiC, in particular, the quality of the material. The high density of defects in 3C-SiC is the consequence of the large mismatches in lattice parameters (19% at RT) and in thermal expansion coefficients (~23% at deposition temperatures and 8% at RT) between 3C-SiC and silicon [4]. After the growth, the stress generated during the cooling phase is tensile. This causes an upward bending (concave) of the wafer and, often, the generation of cracks in the epitaxy and in the substrate. The deflection depends on the crystal and substrate size, the thickness of the epitaxy and of the Si substrate, and the orientation of the epitaxial crystal and the substrate [5]. Different approaches were developed to try to reduce the density of the defects and the wafer bending effect due to stress. The growth of good-quality 3C-SiC on silicon-on insulator (SOI) has been reported [6,7]. The presence of this layer has proved effective in suppressing the presence of voids in the silicon substrate, as the SiO_2_ acts as a barrier against the diffusion of Si atoms. In addition, the stress in the 3C-SiC layer is reduced due to the softening of the oxide layer at growth temperatures. Other approaches were suggested, such as the use of porous silicon (p-Si) as a substrate [8] or the growth on a SiGe buffer layer or on a silicon pillars [9]. 

Recently, we observed that nitrogen and aluminum doping concentrations have a deep impact on the density and the average length of the staking faults (SFs) that reach the surface and so on during the bending of the wafer [10,11]. The use of complementary analysis techniques and processes is necessary to fully understand the effect of doping on the stress distribution along the c-axis (especially at the interface, where the concentration of defects is highest).

Generally, wafer deformation measurements are conducted via X-ray diffraction (XRD) determination of quality and lattice parameters [12]. In this context, Raman spectroscopy in plan also showed a great ability to characterize the residual stress of micro electromechanical systems (MEMS) [13]. However, the reported techniques allow us to obtain average information on the quality of the deposited film.

Using μ-Raman measurement in the cross-section of the grown 3C-SiC samples, it was possible to observe the dependence of the stress and of the quality of the material as a function of the thickness. The typical Raman signal of 3C-SiC monocrystalline epitaxial film consists in a transverse (TO) and a longitudinal (LO) optical phonon mode. The TO mode is not affected by doping and it provides information about the quality of the crystal lattice as well as its stress field. In particular, the shift in the peak is related to the average stress in the material [14,15].

In this paper, we used micro-Raman spectroscopy in cross-section to investigate the effect of the different doping on the distribution of the stress in the silicon substrate and in the grown 3C-SiC film. A number of 3C-SiC films with a thickness up to 10 μm were grown on Si (100) substrates. To quantify the influence of doping on the stress distribution, samples were non-intentionally doped (NID, dopant incorporation below 10^16^ cm^−3^), strongly n-type doped ([N] > 10^19^ cm^−3^), or strongly p-type doped ([Al] > 10^19^ cm^−3^). In silicon at the interface, the stress is always compressive and the maximum value depends on the thickness of the grown film and on the type of doping. In 3C-SiC, the stress at the interface is always tensile and remains so in the first 4 µm. In the remaining 6 µm, the type of stress varies according to the doping.

## 2. Materials and Methods

A number of 3C-SiC thin films were grown in a horizontal, low-pressure, resistively heated hot-wall CVD system. High-purity (5N) silane (SiH_4_) and propane (C_3_H_8_) were used as principal precursors. Hydrogen (H_2_) was used as a carrier gas and constant nitrogen flux (N_2_) or Tri-Methyl-Aluminum (TMA) as an Al source was introduced within the chamber to dope the samples, for n-type and p-type 3C-SiC films, respectively. Hydrogen chloride (HCl) diluted in H_2_ was introduced into the CVD process to boost the growth rate without increasing the surface defects density and allow the fine adjustment of Al incorporation in p-type films.

The 3C-SiC films with a thickness up to 10 μm were grown on Si (100) substrates. To quantify the influence of doping on the stress distribution, samples were non-intentionally doped (NID, dopant incorporation below 10^16^ cm^−3^), strongly n-type doped ([N^+^] > 10^19^ cm^−3^), or strongly p-type doped ([Al] > 10^19^cm^−3^). One NID sample with a thickness up to 20 μm was grown on Si (100) substrates, and one NID sample with a thickness up to 8 μm was grown on Si (111) substrates. The sample grown on Si (111) turned out to have a lower thickness (8 μm) than the sample grown on Si (100) (10 μm) because, for 3C-SiC on Si (111), an extremely strong concave bow was observed. Wafer cracking during growth/cooling are also possible.

Table 1 shows the list of samples. 

Using μ-Raman measurement in cross-section, it was possible to study the dependence of the stress of the material as a function of the thickness, for both silicon and 3C-SiC. The typical Raman signal of 3C-SiC monocrystalline epitaxial film consists in a transverse (TO) and a longitudinal (LO) optical phonon mode. We analyzed the TO mode because it is not affected by doping and it provides information about the strain field. The shift in the TO Raman peak is related to the average strain in the probed material [14,15].

Micro-Raman maps were acquired at room temperature using an HR800 spectrometer integrated system from Horiba Jobin Yvon (Horiba, Lille, France) in a backscattering configuration. The excitation wavelength was supplied by a 633 nm He-Ne continuous-wave laser that was focalized on the sample by a ×100 objective. The scattered light was dispersed by an 1800 grooves/mm kinematic grating.

For the cross analyses, the cut on the wafer made done parallel to the flat side, along the (110), and placed in a cross under the objective. For each sample, several maps were acquired, each with a size of 10 μm × 30 μm, acquiring both in X and in Y, a spectrum every micron. The values of each single map were averaged along the X axis.

Regarding the bow, it is a parameter that represents the flatness of a wafer. It can be measured when the wafer is kept in a free and unblocked state. The bow was measured at the center point of the wafer, determining the distance from the reference plane. The reference plane was obtained by measuring the focal distance at three points around the edge of the wafer using an optical microscope.

## 3. Results and Discussions

**Figure 1:**
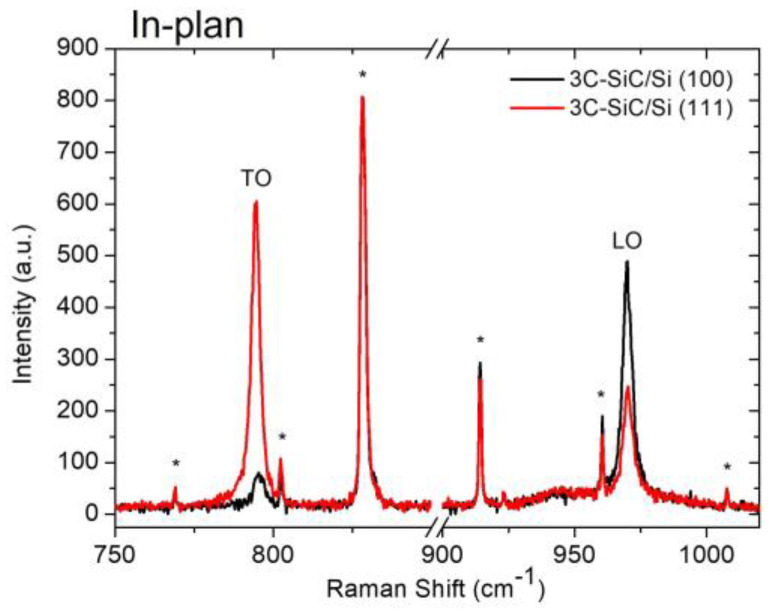
Raman spectra acquired on a 3C-SiC sample grown on Si (100) (sample 142) and one grown on Si (111) (sample O37). The peaks marked with an asterisk belong to the laser and they were used to calibrate the spectra.

In Figure 1, we report the comparison between two Raman spectra acquired on a 3C-SiC sample grown on Si (100) and one grown on Si (111).

It is important to point out that Raman analyses in plan are acquired along the (001). In this configuration, the TO and LO modes are Raman forbidden and active for 3C-SiC grown on Si (100); the TO and LO modes are Raman active and forbidden for 3C-SiC grown on Si (111). Therefore, in the first case, a lower TO peak indicates good crystalline quality; in the second case, a higher TO peak indicates good crystalline quality. These conditions were reversed by placing the samples in a cross.

**Figure 2:**
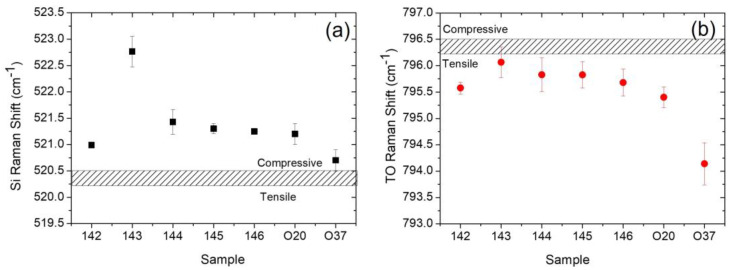
(**a**) Si Raman shift and (**b**) TO Raman shift for all samples. The dashed rectangles enclose the positions of the silicon and of the TO peak commonly reported in the literature for stress-free samples.

Figure 2 shows the Si Raman shift (Figure 2a) and TO Raman shift (Figure 2b) for all samples.

The dashed rectangle encloses the positions of the silicon (Figure 2a) and of the TO peak (Figure 2b) commonly reported in the literature for stress-free samples [14,15,16,17]. The experimental points above the dashed rectangle correspond to a compressive stress; the experimental points under the dashed rectangle correspond to a tensile stress. Raman shift is related to stress by equations reported in the literature for silicon [16] and 3C-SiC [14,15], respectively. However, to estimate the stress on the silicon substrates, we used the calibration curve reported by Yoo et al. [17]. We can observe that all silicon substrates show compressive stress (Figure 2a), while all 3C-SiC films show tensile stress (Figure 2b). Comparing the values for samples 142 and 143, we observe that by increasing the thickness of the 3C-SiC film (10 μm and 20 μm, respectively), the stress increases in the silicon substrate (from about 300 MPa to 900 MPa) and decreases in the 3C-SiC (approximately 258 MPa to almost stress free). Fixing the thickness of the 3C-SiC (10 um) and varying the type of doping (samples 142, 144, 145, 146, and O22) for the silicon substrates, we observe an increase in stress (from 300 MPa to 400 MPa) going from a NID sample (sample 142) to doped samples (samples 144, 145, 146, O22). However, considering the experimental error, the stress in the silicon substrate does not appear to depend on the type of doping. For 3C-SiC films (taking into account the experimental errors), we observe very limited stress variations (about 100 MPa). Finally, comparing the samples grown on Si (100) and Si (111) (sample 142 and O37, respectively), we observe a comparable stress in the Si substrates, and a significantly higher stress (from 200 MPa to 580 MPa) in the 3C-SiC grown on Si (111). The large mismatches in thermal expansion coefficients and lattice parameters (between Si and SiC) create stress in the sample (silicon substrate and SiC film). The stress component due to the different thermal expansion coefficients between the materials always introduces tensile stress. The stress component due to the different lattice parameters instead depends on the experimental conditions, such as the growth rate, the C/Si ratio, and the growth temperature. To try to reduce this component of stress, the system generates several crystallographic defects, the concentration of which is the maximum near the interface and decreases as the thickness of the grown film increases, until it settles at constant values. In particular, stacking faults (SFs) are planar defects produced by either an excess or a lack of a single Si−C bilayer during the growth, and they consist of a wrong stacking order with respect to the 3C-SiC one [18]. Recently, we showed that the density and mean length of the SFs depend on the doping type and concentration [11]. In particular, the presence of high concentrations of nitrogen favors the closure of the SFs.

To observe the dependence of the stress as a function of the thickness in both Si substrate and 3C-SiC film, μ-Raman measurements in cross-section were performed.

**Figure 3:**
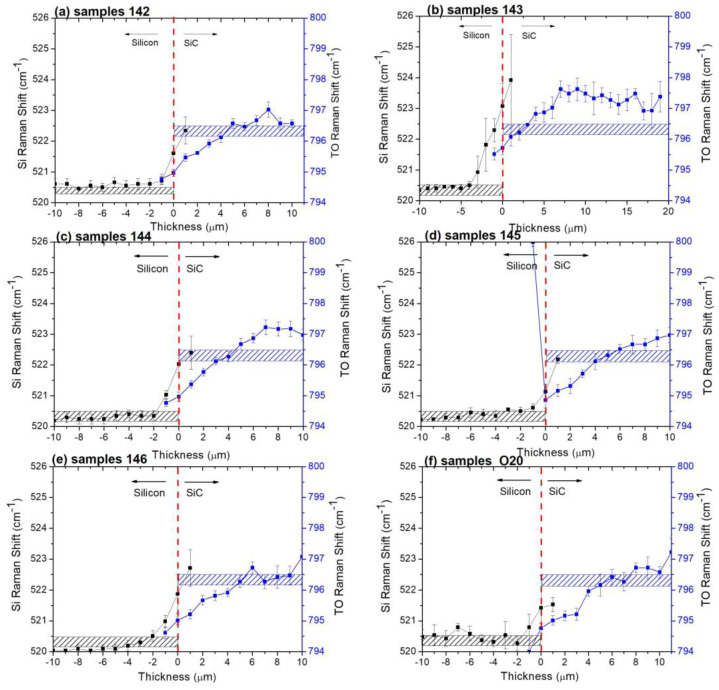
Si Raman shift (black points) and TO Raman shift (blue points) for samples grown on Si (100). In particular, (**a**) sample 142; (**b**) sample 143; (**c**) sample 144; (**d**) sample 145; (**e**) sample 146; (**f**) sample O20. The dashed rectangles enclose the positions of the silicon and of the TO peak commonly reported in the literature for stress-free samples.

Figure 3 shows the Si Raman shift (black points) and TO Raman shift (blue points) for the samples grown on Si (100).

The black and blue points show the trend in the silicon peak and 3C-SiC TO Raman shift, respectively. The dashed rectangles enclose the positions of the silicon (black rectangle) and of the TO peak (blue rectangle) commonly reported in the literature for stress-free samples. The experimental points above the dashed rectangle correspond to a compressive stress; the experimental points under the dashed rectangle correspond to a tensile stress. The Si/3C-SiC interface was set to t 0 (dashed red line). Although the laser spot is about 1–1.5 μm and it is therefore possible to acquire a spectrum for Si or 3C-SiC beyond the interface (point at +1 μm for Si and at −1 μm for 3C-SiC), in the calculations below, we decided to exclude these points of dubious interpretation due to the morphology of the interfaces. Regarding sample 142 (Figure 3a), at the interface, silicon is characterized by compressive stress (about 600 MPa) and then it moves to a stress-free value. The 3C-SiC instead shows tensile stress (about 394 MPa). The stress remains tensile in the first 4 μm; thereafter, it is almost stress free. When increasing the 3C-SiC thickness (sample 143, Figure 3b), silicon is always characterized by compressive stress. However, the stress value is higher at the interface (about 1 GPa) than the previous one, and the stress is deeper inside the silicon wafer (about 3 μm). Instead, 3C-SiC shows a lower tensile stress at the interface (about 200 MPa) and a tensile/stress-free value in the first 4 μm, and then the stress becomes compressive (about 250–300 MPa). By comparing the 3C-SiC trends with the values obtained from in-plan Raman measurements (Figure 2b), it is evident that the in-plan measurements provide an average measurement of the film. In fact, if we compare the average values of the TO position obtained in the plan with the average values obtained from the crosses, we have: 795.6 ± 0.1 (plan) and 796.1 ± 0.4 (cross); and 796.1 ± 0.1 (plan) and 796.2 ± 0.4 (cross), for sample 142 and sample 143, respectively. From the in-plan measurements, increasing the film thickness decreases the stress of the 3C-SiC, in agreement with the literature data [4]. From cross measurements, on the other hand, we observe the evolution of stress inside the film. Studying the stress of films a few microns thick through in-plan analysis to understand what happens at the interface of thicker films is an approximate approach. Comparing the position of the TO at 3 μm from the interface with silicon for a 3 μm thick film (not reported here), a 10 μm thick one (Figure 3a), and a 20 μm (Figure 3b) thick one, we obtain: 795.2 ± 0.2, 795.9 ± 0.1, and 796.5 ± 0.1, respectively.

Figure 3c–f shows the values extracted from the Raman spectra in cross for samples 144, 145, 146, and O22, which are characterized by different doping (see Tab 1).

At the interface, Si always shows compressive stress. However, for samples 144 and 146 (both N^+^-doped at the interface), stress extends deeper inside the silicon wafer than in samples 145 and O22. Moreover, for samples 144 and 146, stress is higher (about 700 MPa) than for samples 142, 145, and O22. Regarding the 3C-SiC layer for all samples, they always show a tensile stress in the first 4 μm, with a value between 382 and 448 MPa at the interface. However, on the surface (considering region between 5 and 10 μm), stress is compressive for sample 144 (about 250–300 MPa) and sample 145 (about 100 MPa), and almost stress free for sample 146 and sample O22.

All results are summarized in Table 2.

From these data, it is evident that the doping variation in the materials has repercussions on all the measured parameters, such as stress in silicon and in SiC, and the bow of the wafers.

Regarding the bow, it is known that strong Al doping leads systematically to concave deformation, while strong N doping results in a convex shape in the wafer. In addition, we have recently demonstrated that high nitrogen concentrations lead to the formation of a high concentration of partial dislocations in 3C-SiC [11]. They delimit SFs, whose development and propagation are suppressed by the presence of nitrogen. The suppression of the SFs leads to a higher bow in the material. In fact, the presence of SFs is essential to compensate for the lattice mismatch between 3C-SiC and Si (5:4 SiC-to-Si lattice plane matching [19]).

Regarding samples 142 and 143, we observe that when increasing the thickness of the film, the bow of the wafer increases (from −50 to −300 μm), probably due to the higher and deeper stress inside the Si substrate (Figure 3a, b). In samples 145 and 146, which show the same bow (400 μm), the thickness of the NID layer present at the interface in sample 145 is probably too small (0.5 μm). Therefore, the amount of nitrogen in the volume of the two samples can be considered equal.

Regarding the stress at the interface, it is always tensile in the 3C-SiC layer, and then it moves to stress-free/compressive stress, and it is always compressive in Si. These trends are in agreement with the model proposed by L. A. Falkovsky, et al. [20]. Using micro-Raman spectroscopy, they investigated both the residual strain and strain relaxation effect in the heteroepitaxial 3C-SiC/Si system. They developed a theory of inhomogeneous shift and broadening for optical phonons, taking into account the phonon interaction with the static strain fluctuations. A first contribution to the phonon frequency shift comes from the averaged strain that exists due to external stress or interface mismatch. This contribution may be positive or negative, depending on whether the stress is compressive or tensile. In the case of a SiC/Si interface, it is tensile in SiC and compressive in silicon. A second contribution comes from the static strain fluctuations due to dislocations, grain or twin structure, and other structural defects. Because all other phonon states are at a lower energy, it is always positive for the top of a phonon singlet (the opposite result would be true for a minimum of the branch). The sum of these two contributions is positive for Si (compressive stress) and negative for SiC (tensile stress), in agreement with our results. Furthermore, because of the different doping influences and the type and density of defects [9,10], this effects the static strain fluctuations and therefore the final stress value.

**Figure 4:**
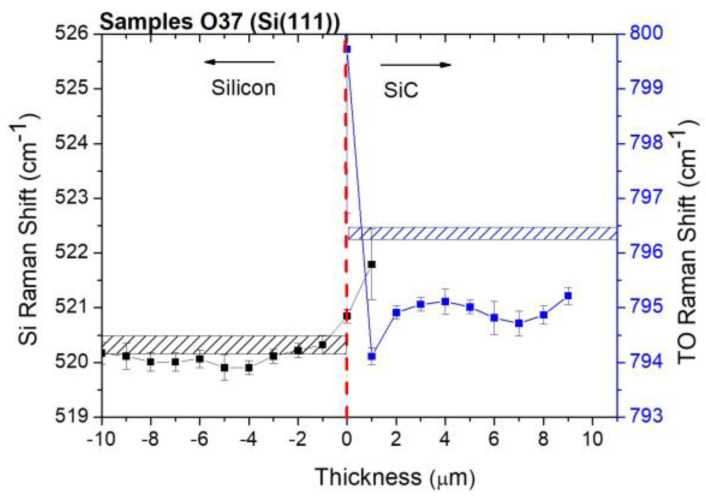
Si Raman shift (black points) and TO Raman shift (blue points) for samples grown on Si (111). The dashed rectangles enclose the positions of the silicon and of the TO peak commonly reported in the literature for stress-free samples.

In Figure 4, we report the Si Raman shift (black points) and TO Raman shift (blue points) for the samples grown on Si (111).

Different trends are observed in both Si and 3C-SiC compared to those reported for films grown on Si (100) (Figure 3). At the interface, Si exhibits a compressive stress of 300 MPa, and then fluctuates between a nearly stress-free/tensile value. On the other hand, 3C-SiC shows compressive stress at the interface (>1 GPa) and then immediately becomes tensile following an oscillating trend (average value 412 MPa). At present, this trend does not appear clear. However, we believe that the high value reported at the interface for the 3C-SiC (the measurement is accompanied by a large experimental error) is an artifact due to the high roughness and defectiveness of the interface to the high bow of the sample, which affects the way it operates in a cross. Regarding the high bow showed by the sample (see Table 2), it is known that for equivalent growth conditions, films grown along the <111> direction on Si (111) tend to store considerably higher tensile residual stress than films grown along the <100> orientation on Si (100) [5]. Moreover, the high tensile residual stress of SiC (111) films only shows a marginal decrease versus thickness. In fact, a higher strain relief efficiency of stacking faults was observed in the SiC (100), which are less steeply inclined (55° and 70° for SiC on Si (100) and Si (111), respectively) onto the growth plane of the epitaxial films. Furthermore, recently, we showed that the typical mechanism valid in (100) growths consisting in the mutual closure of SFs coming from opposing {111} planes that give rise to Lomer and λ-shaped dislocations is replaced by a different kind of evolution. Through high-angle annular dark-field (HAADF)-STEM, we observed an extra plane present inside the crystalline lattice, representing a further partial dislocation surrounding the SF and determining the interruption of its propagation [work under review in microelectronic engineering].

The possibility of understanding how doping influences the distribution of defects [9,10], and therefore of the stress inside 3C-SiC films, is an aspect with important repercussions both in the microelectronics sector and in that of micro electromechanical systems. The crystallographic quality and the excessive curvature of the wafers are two of the aspects that today hold back the development of microelectronic devices. At the same time, recent results on 3C-SiC/Si MEMS resonators have highlighted the relationship between the presence of high tensile stress in the material and the possibility of obtaining very-high-Q-factor resonators [3]. Since the resolution of resonant sensors depends on Q [21], the possibility of obtaining SiC layers with high Young’s modulus and controlled tensile stress is very important because it can allow the fabrication of high-resolution MEMS resonators.

## 4. Conclusions

Doping plays an important role in stress distribution in a 3C-SiC film grown on silicon substrate. Traditional approaches, such as strain gauge, XRD, and Raman analysis in plan allow us to obtain an average value of the stress of the film. This paper describes an example of how this micro-Raman spectroscopy can be applied in cross-section to obtain a distribution of the stress in function of the thickness of the film. 

A number of 3C-SiC films with a thickness up to 10μm were grown on Si (100) substrates. To quantify the influence of doping on the stress distribution, samples were non-intentionally doped (NID, dopant incorporation below 10^16^ cm^−3^), strongly n-type doped ([N] > 10^19^ cm^−3^), or strongly p-type doped ([Al] > 10^19^ cm^−3^). An NID sample was grown also on Si (111).

In silicon, the stress at the interface is always compressive. Once the thickness has been fixed, we observe a stress between 400 and 700 MPa, depending on the doping. For NID samples, on the other hand, a clear dependence on the grown film thickness is observed. Indeed, a stress of 600 MPa and about 1000 MPa was measured for 3C-SiC films 10 µm and 20 µm thick, respectively.

In 3C-SiC, by fixing the thickness (10 µm), we observed that the stress at the interface is always tensile and remains so in the first 4 µm. In the remaining 6 µm, the type of stress varies according to the doping, and it is generally stress free or low-compressive (100–250 MPa).

For equivalent-growth-condition films grown along the <111> direction on Si (111), a considerably higher tensile residual stress is present (412 MPa), and it is not reduced as thickness increases.

## Figures and Tables

**Table 1:** The table shows the list of grown samples with the relative bow. Sample 144 has a 0.5 μm thick N+ layer at the Si interface. Sample 145 has a 0.5 μm thick NID layer at the Si interface.

Sample	Doping (cm^−3^)	Thickness (μm)	Substrate (Si)	Bow (μm)
142	NID	10	(100)	Concave (−50)
143	NID	20	(100)	Concave (−300)
144	N+/NID	10	(100)	Concave (−50)
145	NID/N+	10	(100)	Convex (400)
146	N+	10	(100)	Convex (400)
O20	Al	10	(100)	Concave (−350)
O37	NID	8	(111)	Concave (−1000)

**Table 2:** The table shows the list of all samples, the type of doping, the stress at the interface in silicon (always compressive) and in 3C-SiC (always tensile), the mean stress (compressive) between 5 and 10 μm on the 3C-SiC surface, and the bow of the wafers. (*) In this case, the value is calculated between 5 and 20 μm. (**) The TO measurement is accompanied by a large experimental error, which we believe to be an artifact due to the high roughness and defectiveness of the interface. (***) In this case, the 3C-SiC film is only 9 µm thick.

Sample	Doping (cm^−3^)	Thickness (μm)	Si Stress (MPa)	SiC Stress (MPa)	SiC Stress (MPa) [5–10 μm]	Bow (μm)
142	NID	10	~600	~394	~stress-free	−50
143	NID	20	>1000	~200	~250 *	−300
144	N+/NID	10	~700	~394	~250	−50
145	NID/N+	10	~400	~422	~100	400
146	N+	10	~650	~382	~stress-free	400
O20	Al	10	~500	~448	~100	−350
O37	NID	9	~300	~1000 **	~412 ***	−1000

## Data Availability

The data presented in this study are available on request from the corresponding author.
